# Early postoperative cognitive dysfunction and postoperative delirium after anaesthesia with various hypnotics: study protocol for a randomised controlled trial - The PINOCCHIO trial

**DOI:** 10.1186/1745-6215-12-170

**Published:** 2011-07-06

**Authors:** Federico Bilotta, Andrea Doronzio, Elisabetta Stazi, Luca Titi, Ivan Orlando Zeppa, Antonella Cianchi, Giovanni Rosa, Francesca Paola Paoloni, Sergio Bergese, Irene Asouhidou, Polimnia Ioannou, Apolonia Elisabeth Abramowicz, Allison Spinelli, Ellise Delphin, Eugenia Ayrian, Vladimir Zelman, Philip Lumb

**Affiliations:** 1 Department of Anaesthesiology, Critical Care and Pain Medicine, "Sapienza" University of Rome, Policlinico Umberto I, Rome, Italy; 2 GIMEMA, Data Center, Rome, Italy; 3 Departments of Anesthesiology and Neurological Surgery, The Ohio State University, Columbus, OH, USA; 4 Second Department of Anesthesiology "G.Papanikolaou" General Hospital, Exohi Thessaloniki, Greece; 5 Department of Anesthesiology, Montefiore Medical Center, Albert Einstein College of Medicine, New York, The Bronx, NY, USA; 6 Department of Anesthesiology, University of Southern California, Los Angeles, CA, USA

## Abstract

**Background:**

Postoperative delirium can result in increased postoperative morbidity and mortality, major demand for postoperative care and higher hospital costs. Hypnotics serve to induce and maintain anaesthesia and to abolish patients' consciousness. Their persisting clinical action can delay postoperative cognitive recovery and favour postoperative delirium. Some evidence suggests that these unwanted effects vary according to each hypnotic's specific pharmacodynamic and pharmacokinetic characteristics and its interaction with the individual patient.

We designed this study to evaluate postoperative delirium rate after general anaesthesia with various hypnotics in patients undergoing surgical procedures other than cardiac or brain surgery. We also aimed to test whether delayed postoperative cognitive recovery increases the risk of postoperative delirium.

**Methods/Design:**

After local ethics committee approval, enrolled patients will be randomly assigned to one of three treatment groups. In all patients anaesthesia will be induced with propofol and fentanyl, and maintained with the anaesthetics desflurane, or sevoflurane, or propofol and the analgesic opioid fentanyl.

The onset of postoperative delirium will be monitored with the Nursing Delirium Scale every three hours up to 72 hours post anaesthesia. Cognitive function will be evaluated with two cognitive test batteries (the Short Memory Orientation Memory Concentration Test and the Rancho Los Amigos Scale) preoperatively, at baseline, and postoperatively at 20, 40 and 60 min after extubation.

Statistical analysis will investigate differences in the hypnotics used to maintain anaesthesia and the odds ratios for postoperative delirium, the relation of early postoperative cognitive recovery and postoperative delirium rate. A subgroup analysis will be used to categorize patients according to demographic variables relevant to the risk of postoperative delirium (age, sex, body weight) and to the preoperative score index for delirium.

**Discussion:**

The results of this comparative anaesthesiological trial should whether each the three hypnotics tested is related to a significantly different postoperative delirium rate. This information could ultimately allow us to select the most appropriate hypnotic to maintain anaesthesia for specific subgroups of patients and especially for those at high risk of postoperative delirium.

**Registered at Trial.gov Number:**

ClinicalTrials.gov: NCT00507195

## Background

Postoperative delirium (PD), defined as altered perception with hallucinations, acute decline in cognition and attention and inappropriate behaviour, is a severe and relatively common clinical problem. It develops in up to 56% of the patients (higher risk in elderly patients and after orthopaedic surgery) and is related to an increase in hospital morbidity neuro-functional decline, nursing time per patient, per-day hospital costs, length of hospital stay, rates of nursing home placement and mortality rates [1,2]. Drugs used to induce and maintain anaesthesia can delay postoperative cognitive recovery and favour postoperative delirium. These unwanted effects vary according to each drug's specific pharmacodynamic and pharmacokinetic features and its interactions with the individual patients' characteristics [3,4]. The International Study of Postoperative Cognitive Dysfunction 1 (ISPOCD1), showed that general anaesthesia is related to a postoperative cognitive dysfunction at 1 week in 25.8% and 3 months postoperatively in 9.9% of the patients [5]. The ISPOCD 2 study reported, in 438 elderly patients, no significant differences in POCD between general anaesthesia and local anaesthesia at 3 months follow up (14.3% and 13.9%; p = 0.93) [6]. More recent studies suggest that POCD and PD are more common after general anaesthesia [7]. POCD at 3 months postoperatively also has major long-term consequences including a higher risk of leaving the labour market and dependency on social transfer payments, but also leads to increased mortality at a median 8.5 years [8]. None of these studies have investigated the possible effects on PD of the various anaesthetic/hypnotic regimens used to maintain anaesthesia.

This multicentre, double-blind, prospective, randomized, controlled clinical trial is designed to evaluate PD rate after general anaesthesia maintained with various hypnotics (desflurane, sevoflurane or propofol) in non-premedicated adult patients undergoing surgical procedures other than cardiac or brain surgery. We also aim to test whether delayed postoperative cognitive recovery is related to an increased risk of PD.

Postoperative Delirium and Post anaesthesia Cognitive Recovery Study: PINOCCHIO Trial

Registered at Trial.gov Number: NCT00507195, July 24, 2007

The drugs to be tested in this trial are among the most common anaesthetic hypnotics currently used for maintaining anaesthesia.

### Propofol

Propofol is an intravenous sedative-hypnotic agent used to induce and maintain anaesthesia widely used in neuroanesthesia [9-13]. Propofol infusion induces a dose-dependent vasodilation with arterial hypotension, steady-state propofol blood concentrations are generally proportional to infusion rates [3,9-11]. Propofol reduces cerebral brain flow and cerebral O_2 _metabolism to a similar extent, favourable pharmacokinetics and high-quality recovery profile despite prolonged infusion [12,13].

### Sevoflurane

Sevoflurane has several properties which make it highly useful as a day case anaesthetic. After propofol-induced anaesthesia, awakening is faster from sevoflurane than from isoflurane, faster than after propofol and comparable (in most studies) to desflurane. Subsequent recovery and discharge is generally similar after all these hypnotic agents [14-16]. Sevoflurane-induced anaesthesia is generally well-received and causes fewer side effects than propofol such as hypotension and apnea [9]. When sevoflurane is used for maintenance, the postoperative nausea and vomiting rate is comparable to that for other inhaled anaesthetics, although this complication is more frequent when used for induction. Sevoflurane's tolerability and low solubility make it easier to titrate anaesthesia and may reduce the need for opioid analgesia, thus possibly decreasing the incidence of nausea and vomiting side effects [17-19].

### Desflurane

Desflurane is a halogenated inhalational anaesthetic agent administered by vaporization and used to maintain general anaesthesia. The minimum alveolar concentration (MAC) in oxygen mixture for a 40-year-old-adult is 6% and decreases with age. Despite its high cost, together with sevoflurane, it is gradually replacing isoflurane for use in general anesthesia. It has the most rapid onset and offset of the volatile anaesthetics due to its low solubility in the blood [4,14]. The major drawbacks of desflurane are its low potency and high pungency, and that it tends to induce airway irritability and therefore, is not preferred as an induction volatile anaesthetic. On the other hand, Desflurane presents with the advantage that beyond a cut-off value of 5-6% of inspired concentration, it induces an augmented sympathetic tone with increased heart rate and arterial hypertension, instead of producing a dose-dependent peripheral vasodilatation response [19,20]. Because of the low blood-lipid coefficient that results in limited storage in theadipose tissue and the reduced risk of arterial hypotension, its use might be associated with a lower rate of postoperative delirium [4,18].

### Fentanyl

To minimize confounding variables fentanyl will be used as an opioid for pain management in all study groups. Fentanyl is an half-life opioid analgesic that interacts predominately with the μ opioid-receptor and exerts its principal pharmacologic effects on the central nervous system. Its primary actions of therapeutic value are analgesia and sedation with favorable side-effect profile. Fentanyl may increase the patient's tolerance for pain [19,20].

## Methods

The PINOCCHIO study is a multicentre, double-blind, phase II, prospective, randomized, controlled clinical trial, designed to evaluate postoperative delirium rate after general anaesthesia with various hypnotics in patients undergoing surgical procedures other than cardiac or brain surgery. A secondary end point is to detect whether delayed postoperative cognitive recovery is related to an increased risk of PD.

Enrolment criteria will be assessed in all patients during the routine preoperative assessment and physical examination. After obtaining institutional board approval (IRB), each centre will recruit and randomly assign (with a computerized randomization procedure) patients to one of three treatment arms to receive desflurane, sevoflurane or propofol for anaesthesia maintenance. Patients will be randomly assigned to one of these three strategies with equal probability. Balanced randomization will be maintained at each clinical site using a blocked randomization scheme. Patients will be randomized the day before surgery, once the patient has provided written informed consent and satisfied all the study eligibility criteria. The number of years that patients attended school will be recorded.

### Inclusion and exclusion criteria

Inclusion criteria are age > 18 and < 75 years; American Society of Anesthesiology (ASA) I-III; with no neurological deficits and Glasgow Coma Scale (GCS) Score of 15 [21,22].

Exclusion criteria: Patients with a history of drug abuse, preoperatively foreseen delayed extubation; any condition preventing proper evaluation of cognitive functions such as speech and sensory impairment or psychiatric disorders (language difficulties or psychiatric organic dysfunctions) that could make difficult to administer psychometric tests, will not be enrolled in this study.

To minimize the influence of surgical procedures on postoperative neurocognitive status, patients undergoing cardiac or brain surgery will be excluded. To reduce the risk of PD, premedication benzodiazepine or atropine will be avoided, because the use of these drugs is associated, especially in patients at higher risk, to an excess of PD incidence [23,24]. Premedication could be accomplished with fentanyl (up to 1 mcg/kg) or propofol (up to 0.5 mg/kg). All patients will receive isotonic crystalloid saline solution (5 mL/kg) before anaesthesia induction to minimize the risk of arterial hypotension after anaesthesia induction and will be preoxygenated for 3 minutes with a reservoir bag in 100% O_2 _before intubation to prevent hypoxia. After anaesthesia induction a second intravenous line will be placed for study drug administration purposes. In all patients, anaesthesia will be induced with propofol (2-3 mg/kg IV), fentanyl (2 to 4 μg/kg IV) and non-depolarizing neuromuscular blocking drugs.

After tracheal intubation, patients will be mechanically ventilated with an inspired mixture of air and oxygen (1:1). Ventilation, using a semi-closed breathing system with a fresh gas flow of at least 2 L/min during anaesthesia maintenance, will be adjusted to achieve end-tidal carbon dioxide varying from 30-35 mmHg. No local anaesthesia will be allowed. Anaesthesia will be maintained according to one of the following three study groups: *desflurane + fentanyl*: desflurane will be maintained in a 5.6 to 6.1 MAC intervals and fentanyl (2-3 μg/kg/**h **or 0.7 μg/kg boluses). If necessary, fentanyl (1-2 μg/kg/**h**) can be supplemented just before skin incision; sevoflurane + fentanyl: sevoflurane will be maintained in a 0.75 to 1.25 MAC interval and fentanyl (2-3 μg/kg/**h **or 0.7 μg/kg boluses). If necessary, fentanyl (1-2 μg/kg/**h**) can be supplemented just before skin incision; *propofol + fentanyl*: propofol will be maintained with continuous infusion at 10 mg/kg/h for the first 10 minutes, then reduced to 8 mg/kg/h for the ensuing 10 minutes and reduced to 6 mg/kg/h thereafter. If necessary, fentanyl (1-2 μg/kg/**h**) can be supplemented just before skin incision.

The administration of the assigned hypnotic (desflurane, sevoflurane or propofol) will be stopped at skin dressing. During awakening phase fresh gas flow will be increased to the "minute volume" of the individual patient. When surgery ends, residual neuromuscular blockade will be antagonized with neostigmine 2.5 mg and atropine 1 mg or glycopirrolate at 25% recovery of the first train of four (TOF)-induced twitch and patients were allowed to recover to a TOF ratio of 0.9. Administration of paracetamol (1 g IV) for postoperative analgesia, will be started at surgical wound closure and continued at the dose of 1 g 3 times/day, if necessary opioids can be used to supplement postoperative analgesia.

Postoperative (post-anesthesia) pain will be evaluated every 20 minutes, once patients reach a postoperative Aldrete score ≥ 9, for the first postoperative hour (20, 40, and 60 min) [25]. Research assistants, unaware of the group assignment, will record postoperative pain scores reported by patients during structured interviews using a verbal version of the Visual Analog Scale (VAS), in which a rating of zero corresponds to no pain, ratings of 1-4 indicate moderate pain, and ratings of 5-10 correspond to severe pain [26].

Postoperative (post-anesthesia) cognitive function will be evaluated with 2 test batteries administered individually to all patients at 4 times: before surgery (baseline), and postoperatively-after patients reach a postoperative Aldrete score ≥ 9-every 20 minutes for the first postoperative hour (20, 40, and 60 min). The Short Orientation Memory Concentration Test (SOMCT) is a patient-based test designed to assess cognitive function in terms of level of orientation, memory, and concentration. The SOMCT requires subjects to recall the current year, month, time, a complete sentence, and to repeat in numerical and reverse order the sequence of the months of the year. These 6 variables yield scores varying from 0 to 28, with higher scores indicating better cognitive function [27,28] (Appendix 1, Table 1). The Rancho Los Amigos Scale is a physician-based scale designed to quantify behavioural and cognitive status after acute brain injuries also used in the postoperative intensive care setting. Each state corresponds to 1 of 8 levels, varying from I to VIII, with higher levels indicating better behavioural and cognitive status (Appendix 2, Table 2). Both scales will be evaluated preoperatively and postoperatively by 2 experienced physicians unaware of the group assignment. If the 2 observers' score disagree, the opinion of a third observer will be considered discriminant. The Aldrete score will be assessed by a trained anaesthesiologist, blinded to the allocation treatment group [26]. Every 30-60 seconds the anaesthesiologist will score the patients' activity (ability to move extremities), respiration (ability to cough and breath), circulation (differences in blood pressure from patient's personal baseline), consciousness (ability to keep awake) and skin color (level of peripheral oxygen saturation).

The incidence of postoperative delirium will be evaluated using the Nursing Delirium Scale (Nu-Desc) a tool that can be easily integrated into routine care and clinical practice. The Nu-Desc is a five-item scale that comprises a fifth item for the rating of unusual psychomotor retardation. This scale also takes into account the subjects medical condition (delayed responsiveness, few or no spontaneous actions or words; for example, when the patient is prodded, the reaction is deferred or the patient is unarousable). A positive response to these items brings the maximal screening score to 10. A score of 2 or more on the Nu-Desc correctly identifies the presence of delirium [29,30] (Appendix 3, Table 3). The onset of postoperative delirium will be monitored with the Nu-DeSc every three hours up to 72 hours post anaesthesia.

#### Statistical Analysis

##### Sample Size

This trial has the objective to evaluate if propofol + fentanyl, as well as sevoflurane + fentanyl, is non inferior to desflurane + fentanyl, in terms of PD. To test for non inferiority two comparisons are planned:

✓ propofol + fentanyl vs. desflurane + fentanyl

✓ sevoflurane + fentanyl vs. desflurane + fentanyl

A sample size of 275 patients for each study group, with an allocation ratio of 1:1:1, for a total of 825 patients, is required to achieve 80% power -with a significance level of 0.05- to detect a non-inferiority margin difference between the group percentage of 3% in the primary end-point variable (PD rate). The reference group percentage is 10%. The treatment group percentage is assumed to be 13% under the null hypothesis of inferiority. The power was computed for the case when the actual treatment group percentage is 7%. Sample size calculation has not been corrected for possible multiple comparisons. The statistical test used was the one-sided Score test (Farrington & Manning) [31]. Calculations were implemented in PASS2005 [32].

Patients will be randomly assigned in consecutive order in allocation blocks. No patient stratification will be done. Dunnett's test will be used after ANOVA to test the null hypothesis that no group has its mean significantly different from the mean of the reference group [33].

### Ethical considerations

The Pinocchio study will be conducted according to the principles of the Declaration of Helsinki (version of 2004). The Medical Ethics Committee of the Policlinico Umberto I approved the protocol before start of the trial (May 18, 2007 N 426/07; President Prof Aldo Isidori).

Approval by the local medical ethical review board is required for each participating centre before start of inclusion. In advance of the trial starting at a site the Principal Investigator must agree to adhere to Good Clinical Practice Guidelines and all relevant regulations in their country.

## Results

The first patient was recruited in September 2010. Currently 2 centres have obtained local IRB approval. To date (April 2011) 269 patients have been enrolled in the study.

## Discussion

The results of this multicentre, double-blind, prospective, randomized, controlled clinical trial will help anaesthesiologists to be able to choose the hypnotic drug least likely to induce PD after general anaesthesia for surgical procedures other than cardiac or brain surgery. By clarifying the influence of PD on early postoperative cognitive impairment our findings should also help tailor hypnotic-anaesthesiological strategies to the individual patient. The subgroup analysis used to categorize patients according to demographic variables relevant to the risk of PD (age, sex, body weight) and to the preoperative score index for delirium, should help to defined the risks of PD in the various subgroups.

We sought that excluding patients undergoing surgical procedures other than cardiac or brain surgery will minimize the influence of hemodynamic variablitiy and the surgical procedure per se on the postoperative neurocognitive status of the patients. The risk of PD can be predicted and effective strategies exist to reduce this risk. In 126 patients undergoing hip fracture repair to receive usual care alone or supplemented by several additional measures (including: supplemental oxygen during surgery, optimizing electrolytes and blood glucose preoperatively, discontinuing high-risk medications, maintaining adequate nutritional intake, encouraging patients to get out of bed on the first postoperative day, treating severe pain), reduce delirium rate from 50% to 32% in the intervention group and severe delirium rate from 29% to 12% [34].

Several drugs can increase the risk of PD and worsen postoperative cognitive recovery. Perioperative atropine and other drugs with anticholinergic properties, ie, benzodiazepines and the opioid agent meperidine increase the risk of PD. In general, the older the patient, the less appropriate are these agents [24]. Many drugs not typically recognized as anticholinergics, including tricyclic antidepressants, first-generation antihistamines and high-dose H_2_-receptor blockers may have potent anticholinergic activity therefore these agents too should be avoided in elderly patients [35]. Postoperative impairment of memory and concentration has been attributed to benzodiazepines in elderly patients undergoing cataract surgery, with a statistically significant correlation between the extent of impairment in the memory performance and amount of nitrazepam given during the postoperative week [36].

Previous reports suggest as significant risk factors for PD, increasing age, blood urea levels, cardiothoracic index, hypertension, smoking habits, blood replacement during bypass, atrial fibrillation (AF), pneumonia, and postoperative blood fluid balance. These reports also include prior cognitive impairment and reduced hemoglobin and hematocrit levels [37,38]. Preoperative fluid fasting is an independent risk factor for early PD and dehydration is a known risk factor for delirium [39,40]. To minimize this relationship, during anaesthesia induction, patients will receive isotonic crystalloid saline solution (5 mL/kg) through a peripheral intravenous catheter.

For PD screening we used the Nu-Desc which assesses five signs or symptoms: orientation, behaviour, communication, illusions and psycho-motor retardation (Appendix 1, Table 1). Each sign or symptom is rated on a three-point scale, from 0 (indicating the absence of the described sign or symptom) to 2 (indicating that the sign or symptom fulfils all the diagnostic criteria) [29]. According to this scale a cumulative score of ≥ 2 is diagnostic for PD. The Nu-Desc has high diagnostic specificity and excellent sensitivity and is the most appropriate scale for the perioperative setting [30].

In conclusion, the results of this comparative anaesthesiological trial should allow us to detect whether the use of each of the three hypnotics tested is related to a significant difference in PD rate. This information could ultimately help in selecting the most appropriate hypnotic to maintain anaesthesia in a specific subgroup of patients and especially those at high risk for PD.

## Abbreviations

PD: Postoperative Delirium; POCD: Postoperative Cognitive Dysfunction; MAC: Minimum Alveolar Concentration; IBA: Institutional Board Approval; ASA: American Society of Anesthesiology; GCS: Glasgow Coma Scale; Nu-Desc: Nursing Delirium Scale; SOMCT: Short Orientation Memory Concentration Test; RLAS: Rancho Los Amigos Scale; AF: Atrial Fibrillation.

## Competing interests

The authors declare that they have no competing interests.

## Authors' contributions

FB, the principal investigator, is responsible for coordinating the PINOCCHIO study. All authors contributed to the design of the study and to draft the manuscript and approved the final version. FP is responsible for statistics and data analysis. All authors will participate in interpretation of results.

## Appendix

Appendix 1

**Table 1:** The Short Orientation Memory Concentration Test

What is the current year?	Correct answer scores: 4 Incorrect answer score: 0
What is the current month?	Correct answer scores: 3 Incorrect answer score: 0

What time is it?	Correct answer scores: 3 Incorrect answer score: 0

Count backwards from 20 to 1	Max score 4

Say the months of the year backwards	Max score 4

Repeat the information given in the preceding sentence	Max score 10

Appendix 2

**Table 2:** The Rancho Los Amigos Scale (RLAS)

Level I	No response to pain, touch, sound or sight
Level II	Generalized reflex response to pain

Level III	Localized response. Blinks to strong light, turns toward/away from sound, responds to physical discomfort, inconsistent response to commands

Level IV	Confused/Agitated. Alert, performs motor activities but behavior is non purposeful, extremely short attention span

Level V	Confused/Non agitated. Gross attention to environment, highly distractible, inappropriate verbalization

Level VI	Confused/Appropriated. Inconsistent orientation to time and place, consistently follows simple directions

Level VII	Automatic/Appropriated. Skills noticeably deteriorated

Level VIII	Purposeful/Appropriate

Appendix 3

**Table 3:** The Nursing Delirium Screening Scale (Nu-DESC)

	Symptoms	Symptoms Rating 0 = no symptoms 1 = mild symptoms 2 = pronounced symptoms
1	**Disorientation** Verbal or behavioural manifestation of not being oriented to time or place or misperceiving persons in the environment.	

2	**Inappropriate behaviour** Behaviour inappropriate to place or for the person; e.g., pulling at tubes or dressings, attempting to get out of bed when that is contraindicated, and the like.	

3	**Inappropriate communication** Communication inappropriate to place or for the person; e.g., incoherence, noncommunicativeness, nonsensical or unintelligible speech.	

4	**Illusions/Hallucinations** Seeing or hearing things that are not there; distortions of visual objects.	

5	**Psychomotor retardation** Delayed responsiveness, few or no spontaneous actions/words; e.g., when the patient is prodded, reaction is deferred or the patient is unarousable	

	**Total Score**	

	**Delirium**	**≥ 2**
